# News and Miscellany

**Published:** 1904-02

**Authors:** 


### News and Miscellany.

In Memory of Dr. H. A. West.—At the regular meeting of the Fayette County Medical Society the following resolutions were offered and accepted:

*Resolved,* That in the death of Dr. H. A. West, not only the medical profession of Texas, but that of the United States, has lost one of its brightest members.

*Resolved,* That the Fayette County Medical Society extend its sympathy to the bereaved family.

*Resolved,* That a copy of these resolutions be sent to the bereaved widow, and for publication copies be sent to both the *Texas Medical News* and the Texas Medical Journal.

H. A. Tutwiler, President,
I. E. Clark,
F. H. Neuhaus,
P. Chapman, Secretary,
Committee.

Ho for St. Louis.—Take the "Texas Railroad," the I. &. G. N. Shortest and quickest and best route.

For Denver and the Panhandle. You'll want to go to Denver this summer. See the new ad. of the Fort Worth and Denver Road and write to Mr. Glisson, the General Passenger Agent, about it.

Dr. T. J. Bell, of Tyler, has been appointed by the Governor a member of the State Board of Medical Examiners, vice Dr. J. C. Jones, deceased. This is an excellent appointment and most worthily bestowed. It will give universal satisfaction to the members of the State Medical Association.

Dr. J. W. Talbot, Texarkana, Texas, will receive for treatment and give personal attention to cases of morphine, cocaine and chloral addiction.

Will Repair Your Electrical Machines.—Static and all electrical medical apparatus put in running order. I am also agent for electrical and X-ray apparatus. Oliver Brush, 710 Colorado Street, Austin, Texas.

New Orleans Polyclinic.—Seventeenth annual session opens

November 2, 1903, and closes May 28, 1904. Physicians will find the Polyclinic an excellent means for posting themselves upon modern progress in all branches of medicine and surgery. The specialties are fully taught, including laboratory work. For further information, address New Orleans Polyclinic, postoffice box 797, New Orleans, La.

---

"Croup kills" "Calcidin Saves Life." See Dr. Abbott's new ad. under "Contents," and write for trial box.

---

Dr. J. D. Beck, of Mason, died of Bright's disease at his home January 25th ult. He was a graduate of Tulane University, class of 1870. Dr. Beck was one of the most prominent and able physicians in his section of the State, and had lived in Mason twenty-five years.

---

Dr. Jno. C. Jones died at his home in Gonzales, Texas, January 21st ult. Dr. Jones was a graduate of the University of Edinburg, Scotland, class of 1860. He was a member of the State Board of Medical Examiners of Texas, and in 1885 was Vice President of the Texas State Medical Association. He was a distinguished Confederate surgeon, having been Hood's medical director during the Civil War. Dr. Jones was in his sixty-seventh year. He was a native of Alabama and came to Texas after the war.

---

For Sale.—Drug store and home of a deceased physician. Large practice for capable German physician in a large German neighborhood. Former doctor's cash practice amounted to over $3000 per annum. For particulars write to Judge S. R. Blake, Bellville, Austin County, Texas.

---

Pretty Christmas Party to Employes.—One of the pleasantest events of the holiday season in the drug circles was a buffet luncheon given on last Thursday by Fairchild Bros. & Foster to their employes. It was a complete surprise to the guests, who numbered about 140. A big table in the work room on the fourth floor at 74 Laight street fairly groaned with all the good things prepared by Maresi's chefs, and was decorated with potted plants presented to members of the firm by the members of the office staff.

B. T. Fairchild welcomed the guests to the "Christmas Party," as it was called, and E. W. Dusenberry responded, after which Messrs. S. W. Fairchild and M. G. Foster also spoke. There was music during and after the luncheon, which began at 12:30, and at three o'clock the annual presents in gold were distributed among the employes and the rest of the day was declared a holiday.

---

AT THE CLOSE OF DAY.

If I can say at close of day,
That I have had one kindly thought,
That I one kindly deed have wrought,
Or any kindly lesson taught
By spoken word;

If I have brought one gleam of light,
If I have changed one wrong to right,
If I one suffering cry tonight
In pity heard.

The day has not been spent in vain,
Though filled with grievous care and pain;
That thought, and word and deed remain
To bless my way, at close of day!

—*Emily H. Watson.*

---

DR. H. W. DUDLEY, of Hillsboro, died in that city January 21st ult., aged 49. Dr. Dudley was a native of Kentucky and was a popular and well known member of the Texas State Medical Association. He had lived in Texas about twenty years.

---

GEORGE P. HACHENBERG, M. D., New York University, 1851, died at his home near Austin, Texas, January 8, aged 80. Dr. Hachenberg was at one time a United States army surgeon. He is said to have been the originator of the telephone idea, and published some twenty-five years ago a magazine article on a "musical telegraph."

---

DEATH OF MRS. J. D. OSBORNE.—Mrs Julia Pitts Osborne, wife of Dr. J. D. Osborne, of Cleburne, Texas, and mother of Drs. E. B. Osborne and J. D. Osborne, Jr., died at her home January 17th ult. Mrs. Osborne was a public spirited and useful woman, a real philanthropist, whose good deeds will be long remembered and whose loss deplored by the people of Cleburne.

---

VICTOR KOECHL & Co., the well known importers and sole licensees for the United States of the products of the Farbwerke vorm Meister Lucius & Bruning, and other famous German pharmacal houses, sends us an "acute poisoning chart," which they have recently gotten up. It will be found useful for reference by practicing physicians. A copy may be had for the asking, if the *Red Back* be mentioned.

---

Christian Science Diagnosis.—Christian Science Mother—Eleanor, what is the matter? Christian Science Child—Oh, mamma, I got a terrible error of the mind in my stomach.—*New York Life.*

---

A party of health officials went to Mexico February 1st inst., to inspect the sanitary condition of that country, with a view of getting the Mexican government to institute measures of prevention of yellow fever in future. This is in pursuance with an agreement that it is understood was entered into between the Mexican health officials and Surgeon General Wyman recently. The party consisted of Health Officers Taber of Texas, Saunders of Alabama, Purnell of Mississippi, Souchon, Nolte and Owens of Louisiana, Wurtenbaker of the United State Marine Hospital Service, and McKnight of Laredo. Mrs. Tabor accompanied the party.

---

Railroad and Sleeping Car Disinfection.—The date when the rules and regulations promulgated by State Health Officer Tabor (see Texas Medical Journal for January) will be enforced for carrying into effect the new Texas sanitary law, has been changed from February 11th (inst.), to March 31st, by request of certain railroad and Pullman officials.

---

The cost of the recent epidemic of yellow fever at Laredo, according to the report of State Health Officer, was $4012.19. Including this sum, the total amount expended along the Rio Grande on account of yellow fever (September, October and November, 1903), was $6080.33.

---

If chemistry is the mathematics of medicine, then the proper use of serum is the most positive thing in therapeutics.

Roux, the eminent disciple of Pasteur, says: "Give not less than 2500 units of pure antitoxin." By pure antitoxin he means a serum that does not need an antiseptic to preserve it. The antitoxin made by Roux neither needs nor contains any preservative agent. The Pasteur methods of manufacturing antitoxin preclude the necessity for the admixture of any foreign agent like trikresol. Pure antitoxin is harmless. One can not give an overdose. Roux warns against giving an underdose. Based upon his vast clinical experience he thinks it almost criminal to temporize by giving a tentative dose. As 2500 units is the proper dose, the antitoxin made by Roux at the Pasteur Institute in Paris, France, is put up only in 10 c. c. vials of 2500 units. Roux recognizes but one standard. The same serum is supplied in a dry form that is perfectly soluble and that keeps for years without any deterioration.

Every doctor in Texas ought to have at least one tube on hand. "In time of peace prepare for war."

